# Ethical analysis of childhood vaccination information processes among Turkish parents in the United States

**DOI:** 10.3389/fpubh.2026.1926381

**Published:** 2026-09-18

**Authors:** Emre Çelik, İbrahim Topçu, Perihan Demir, Beyzanur Kaç, Eray Serdar Yurdakul

**Affiliations:** 1 Department of Pediatrics, Bursa City Hospital, Bursa, Türkiye; 2 Department of Medical History and Ethics, Hamidiye Faculty of Medicine, Health Sciences University, Istanbul, Türkiye; 3 Department of Medical History and Ethics, Hamidiye Institute of Health Sciences, Health Sciences University, Istanbul, Türkiye; 4 Department of Medical History and Ethics, Gülhane Faculty of Medicine, Health Sciences University, Ankara, Türkiye

**Keywords:** bioethics, childhood vaccination, cultural competence, immigrant parents, informed consent, Muslim minority, qualitative research, vaccine hesitancy

## Abstract

**Introduction:**

Highly educated, established immigrant populations remain understudied in vaccine-hesitancy research, which has focused primarily on refugee and low-literacy samples. This study examines how foreign-born Muslim Turkish parents in the US experience informed consent for childhood vaccination within a legal context that treats vaccination as a mandatory condition of school enrollment.

**Methods:**

A qualitative descriptive design was employed using semi-structured in-depth interviews with 13 foreign-born Turkish Muslim parents recruited via snowball sampling across five U. S. states. Data were analyzed using codebook thematic analysis within Braun and Clarke’s six-step framework. After two coders performed manual coding independently, Google NotebookLM was integrated for preliminary code extraction. A third author participated in the comparative evaluation and code book consensus, and the findings were organized into themes.

**Results:**

Participant-reported experiences yielded four main themes: (1) Technical language and understandability barriers in the information process; (2) Ethical competence and barrier management in clinical communication; (3) Vaccine mandates in education and enrollment processes and their impact on the informed consent process; (4) Information verification mechanisms.

**Discussion:**

Participants described mandatory school immunization requirements as shifting clinical consent encounters toward procedural compliance. Independent of the mandate, clinical encounters did not equip parents to discuss vaccine ingredients, and unaddressed religious inquiries prompted digital and community-based verification. Formal religious guidance on these concerns exists in the US but appeared unfamiliar to participants, unlike in some Muslim-majority contexts with coordinated religious-regulatory guidance. These findings, from a small qualitative sample, suggest that clinical communication alone may not resolve parents’ difficulties, pointing to possible value in broader coordination among healthcare systems, manufacturers, and religious authorities.

## 1 Introduction

Childhood vaccinations are considered one of the most fundamental building blocks of public health, preventing approximately 2 to 3 million deaths worldwide each year (1, 2). However, the increasing global vaccine hesitancy currently constitutes a central crisis area in terms of bioethics and health communication (3, 4). Informed consent plays a vital role in overcoming this crisis; it is a trust-building and dynamic communication process between the physician and the family, based on the criteria of intention, understanding, and voluntariness (5, 6).

Studies in the current literature have mostly focused on the vaccination attitudes of immigrant parents and the barriers to accessing the system (7, 8), and even within this literature, the populations most often studied are recently arrived, low-literacy, or refugee samples rather than established, highly educated immigrant communities (7–9). The ethical dimension of the informed consent process for this latter group—who navigate the same mandatory system but do not share the knowledge gaps and access barriers most commonly studied—remains largely unexamined.

In the United States, compliance with routine childhood vaccination is not simply recommended but is enforced as a formal condition of school enrollment, and the specific way this requirement is administered varies meaningfully by state. In Connecticut, where the majority of our participants resided, state law requires documented proof of vaccination for school entry and, since the 2022–23 school year, no longer permits religious exemption as a basis for waiving this requirement, so that non-compliance can result directly in a child’s exclusion from school (10). Pennsylvania, where one participant resided, imposes the same school-entry documentation requirement but continues to recognize a religious or moral-conviction exemption in statute (11, 12). This legal architecture reflects a broader national shift: although 45 states still permit religious exemptions and 15 also allow personal belief exemptions, a small but growing number of states—including Connecticut, along with New York and Maine—have eliminated religious exemptions altogether over the past several years, a change the American Academy of Pediatrics has actively supported (13, 14). The constitutional basis for treating vaccination as a condition of school attendance dates to Jacobson v. Massachusetts (1905) and was extended to the school-entry context in Zucht v. King (1922); more recent public health ethics scholarship argues that removing exemptions is justifiable only when a vaccine-preventable threat is genuinely imminent and no less restrictive alternative would achieve comparable protection, since exclusion from school carries consequences that extend well beyond the vaccination decision itself (15). Sophian et al. (16) suggest that such policy changes in the US are read internationally less as evidence-based public health decisions than as signals about institutional trust, with implications extending beyond US borders. It is against this specific regulatory backdrop—vaccination as an enforceable precondition for schooling rather than a voluntary recommendation—that the informed consent experiences described by parents in our study must be understood.

These experiences, however, are not reducible to a single phenomenon. At least three analytically distinct difficulties are frequently conflated in the literature and, we argue, in the framing of this study to date. The first concerns the adequacy of the consent process itself—whether parents received sufficient, comprehensible information to meet the criteria of intentionality, understanding, and voluntariness a valid consent requires (5, 6). The second concerns coercion that follows from the mandatory structure of the requirement, independent of how well the parent was informed: a vaccine mandate is, as Williams (17) argues, a form of government coercion that limits parental autonomy by attaching a cost—here, exclusion from school—to non-compliance, defensible only when proportionate to a grave threat and when no less restrictive alternative exists. A fully informed parent may still experience the encounter as coercive simply because refusal is not a real option. The third concerns a broader mistrust of the healthcare system that is not specific to the vaccination encounter at all, and reflects the relationship between immigrant communities and host-country institutions more generally; this is the dimension most emphasized in the existing literature on refugee, immigrant, and migrant populations, which attributes mistrust to knowledge gaps, limited access to care, and prior negative institutional experience (9). This distinction matters: the first difficulty can in principle be addressed through better communication, the second requires reconsidering the mandate’s structure, and the third requires institutional trust-building beyond any single clinical encounter.

Building on this framework, the present study examines how Turkish parents living in the US experience the informed consent process for their children’s routine vaccinations, and how the pressures created by mandatory vaccination requirements (5, 18) intersect with their understanding of, and voluntary agreement to, vaccination. In doing so, the study aims to address a gap in the literature: the informed consent experiences of a minority population that does not fit the profile most commonly studied in this area, examined here from both a clinical and an ethical perspective.

## 2 Materials and methods

### 2.1 Research design and study population

This qualitative research examines the knowledge and attitudes of Muslim Turkish parents living in the US regarding childhood vaccinations and specifically aims to investigate and ethically analyze the information processes they experienced during vaccination periods. For this purpose, an exploratory qualitative design employing codebook thematic analysis was adopted, integrated with a normative bioethical analysis framework (19).

All participants in this study were foreign-born Muslim Turks who migrated from Turkey to the United States (residing in the US between <5 and >25 years) across five U. S. states: Connecticut (*n* = 7), Massachusetts (*n* = 2), New Jersey (*n* = 2), Pennsylvania (*n* = 1), and Florida (*n* = 1). Of the 12 participants whose educational attainment was recorded, all held at least a bachelor’s degree (six held a doctoral degree, one held a master’s degree, and five held a bachelor’s degree); educational background for P6 was unreported. Participants were selected using the snowball sampling method. This method aims to reach individuals one by one, starting with the person who can provide the most information on the subject, in situations where it is difficult to reach all segments of the population (20, 21). The first participant in the study was a person whom the fifth author, who was a researcher at the University of Connecticut at the time the study was designed, met there; the other participants were identified through snowball sampling.

The criteria for parents included in the study were: living in the US, being Turkish and Muslim, and having a child of vaccination age. To establish explicit inclusion parameters, a ‘child of vaccination age’ was defined as having at least one child between 0 and 18 years of age (covering the pediatric spectrum from infancy through high school completion/Grade 12). Eligibility was grounded in both the CDC’s Routine Childhood Immunization Schedule and state-specific mandatory school/daycare entry requirements. This aimed to ensure participants had experience with informing people about childhood vaccinations. Exclusion criteria included being non-Turkish and non-Muslim and not having a child of vaccination age while living in the US, due to their incompatibility with the research population. The average age of the 13 eligible volunteer participants was 38.23. One participant held a master’s degree, six held doctorates, and the minimum educational level was a bachelor’s degree. All participants were first-generation immigrants born in Turkey who relocated to the United States (none were US-born), with durations of residence ranging from under five years to over twenty years. Demographic information about the participants is presented in Table 1; however, to mitigate the risk of identifying the participants, their ages and the number of years they have lived in the United States are presented as ranges rather than exact figures.

**Table 1:** Demographic characteristics of the participants.

Participant (P)	Gender	Age	Location	Education level	Duration of residence in the US	Number of vaccinated children/children navigating immunization schedule
P1	Male	41–45 years	Connecticut	Bachelor’s	≥ 20 years	3 + children
P2	Male	31–35 years	Connecticut	Bachelor’s	≥ 20 years	1–2 children
P3	Male	41–45 years	Connecticut	Master’s	≥ 20 years	3 + children
P4	Male	31–35 years	New Jersey	Doctorate	≤ 10 years	1–2 children
P5	Male	46–50 years	Pennsylvania	Doctorate	10–20 years	1–2 children
P6	Male	46–50 years	Massachusetts	Unreported	≥ 20 years	1–2 children
P7	Male	46–50 years	Connecticut	Doctorate	10–20 years	3 + children
P8	Male	61–65 years	New Jersey	Bachelor’s	≥ 20 years	3 + children
P9	Male	36–40 years	Massachusetts	Doctorate	10–20 years	1–2 children
P10	Male	36–40 years	Connecticut	Doctorate	10–20 years	3 + children
P11	Female	41–45 years	Connecticut	Bachelor’s	10–20 years	1–2 children
P12	Female	26–30 years	Connecticut	Bachelor’s	≤ 10 years	1–2 children
P13	Male	36–40 years	Florida	Doctorate	10–20 years	1–2 children

US, United States.

### 2.2 Data collection method

Data were collected in January 2026 via the Zoom video conferencing platform using a semi-structured interview guide specifically developed for this study. The research team designed this tool based on current literature regarding informed consent, vaccine attitudes (2, 5, 22, 23). The interviews were conducted in Turkish—the native language of both participants and researchers—by the first, third, and fourth authors, who had never met or interacted with the participants before. The online interviews were audio-recorded with participants’ informed consent. Sample size determination was guided by codebook thematic analysis principles, focusing on conceptual depth and code saturation. In successive interviews, data collection was monitored not merely for thematic repetition but for conceptual granularity and depth. Data collection concluded after the 13th interview, at which point code saturation was achieved, as no novel codes or thematic dimensions emerged from the analysis. The interviews with 13 participants lasted an average of 32.5 min. The audio recordings were listened to attentively by the authors and transcribed verbatim in Turkish, after being anonymized by removing identifying information to preserve the verbal statements and confidentiality. These complete participant transcripts were systematically reviewed and audited prior to data analysis, and selected direct quotations were translated into English by research.

### 2.3 Data analysis

Qualitative data were analyzed using a codebook thematic analysis approach operationalized within Braun and Clarke’s six-step framework (24). The analytical process combined independent manual human coding with AI-assisted preliminary code extraction, structured across six sequential steps as follows:

Step 1 Data Immersion and Anonymization: Prior to analysis, all audio recordings were verbatim transcribed and fully anonymized by assigning participant codes (K1–K13) to protect confidentiality. Coders engaged in repeated reading of the transcripts to achieve deep immersion in the data.

Step 2 Initial Code Generation and Parallel AI Extraction: Two coders (P. D. and B. K.) conducted manual coding fully independently. These human coders completed their initial immersion and independent code generation prior to reviewing the AI-generated outputs and prior to seeing each other’s coding results. In parallel, all 13 anonymized transcripts were imported together and analyzed collectively as a complete dataset (rather than separately transcript-by-transcript) into Google NotebookLM (powered by the Gemini 1.5 Pro language model architecture) to extract preliminary descriptive code candidates using a standardized analytical prompt (“Act as an expert qualitative researcher. Analyze the following interview transcript to identify descriptive codes and recurring patterns, regarding participants’ experiences, knowledge, attitudes, and information processes concerning childhood vaccinations and informed consent”).

Step 3 Evaluation, Comparison, and Code Selection: To ensure analytical rigor and eliminate individual bias, a third author (İ. T.) joined the primary coders (P. D. and B. K.) to systematically compare and evaluate the independent human codes alongside the AI-extracted codes. Selection was guided by two criteria: (a) empirical fidelity to the verbatim data and (b) conceptual clarity and accuracy of expression. Where a human-generated manual code demonstrated greater fit with the empirical data or captured the contextual nuance of participant narratives more effectively, the human manual code was prioritized over the AI suggestion. Unclear, overly broad, or unverified AI codes were discarded.

Step 4 Codebook Construction: Verified human codes and refined AI-supported codes were synthesized into a structured codebook with explicit operational definitions. Discrepancies between coders and discrepancies between human vs. AI codes were resolved through multi-author cross-verification meetings (involving P. D., B. K., and İ. T.) rather than statistical inter-coder reliability metrics.

Step 5 Developing and Reviewing Themes: Codes from the finalized codebook were iteratively sorted and clustered into potential thematic patterns. The candidate themes were cross-checked against the entire dataset to ensure coherence and empirical saturation.

Step 6 Finalizing the Thematic Architecture and Reporting: The final thematic structure was defined, named, and organized to construct the qualitative narrative presented in the Results section. The human-AI collaborative thematic analysis workflow, illustrating the integration of manual independent coding with Google NotebookLM code extraction and third-author consensus, is depicted in Figure 1.

**Figure 1:**
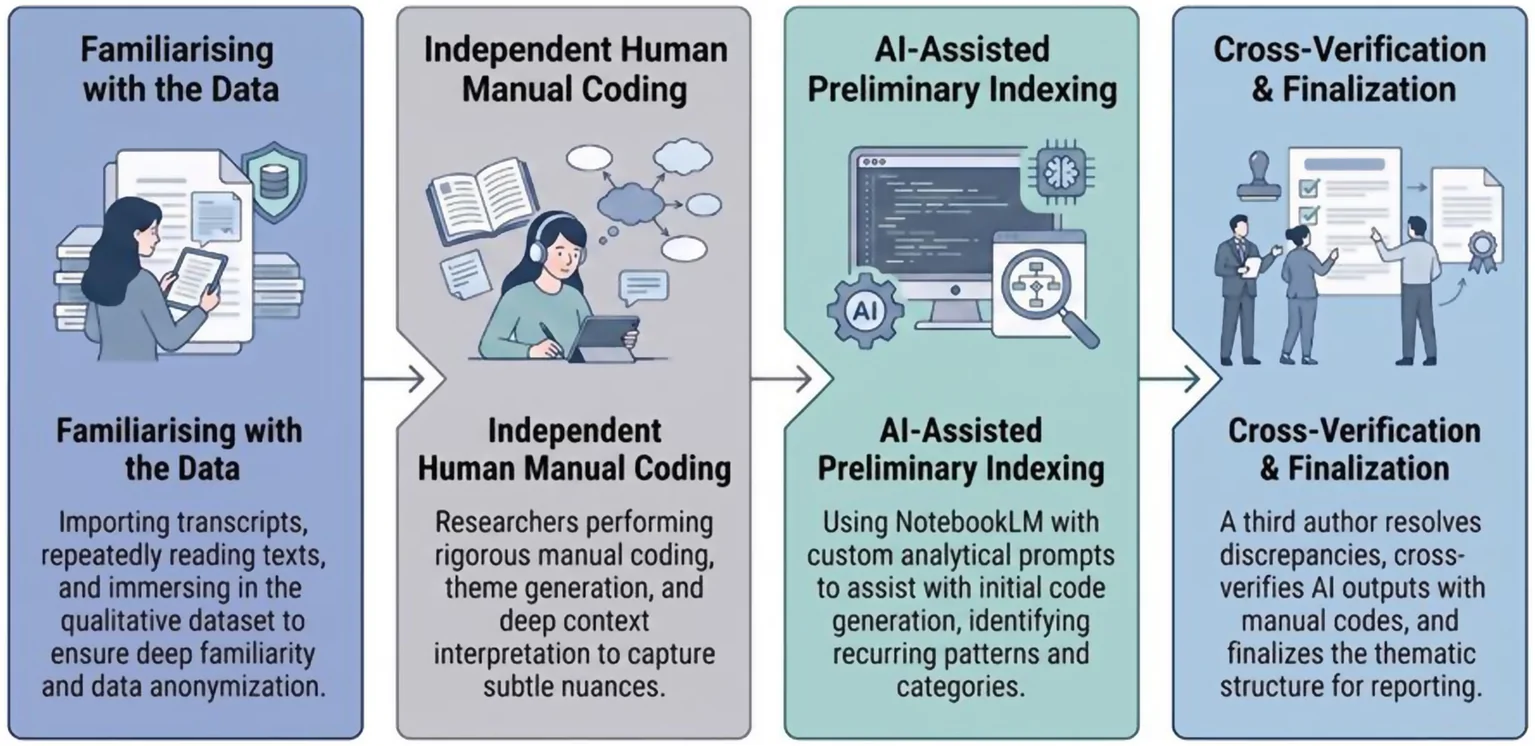
Qualitative data analysis process.

#### 2.3.1 Ethical analysis framework

To perform the ethical analysis of the qualitative data, an applied bioethics framework was operationalized alongside the codebook thematic analysis. Qualitative findings were systematically appraised against Faden and Beauchamp’s model of autonomous decision-making in informed consent, evaluating the three core criteria: intentionality, understanding, and voluntariness (5). Furthermore, the tension between parental autonomy and state-mandated vaccination policies was analyzed using Beauchamp and Childress’s principles of biomedical ethics (specifically autonomy versus paternalism/coercion) and Savulescu’s four-condition framework for mandatory vaccination (25). Empirical codes and themes derived from participant narratives were iteratively mapped onto these normative bioethical dimensions to critically evaluate the moral adequacy of clinical communication and public health mandates.

### 2.4 Limitations

This study has several limitations tied to its recruitment strategy. Participants were identified through snowball sampling beginning from a single contact in the fifth author’s professional network at the University of Connecticut (Section 2.1); as a result, the sample is considerably more educated—six of thirteen participants held doctorates—and more university- and professionally-connected than the broader Turkish Muslim population in the US. Furthermore, this sample raises the possibility that participants share the same or interconnected social or professional Turkish Muslim backgrounds. This bears on transferability rather than on the validity of what was found: it should not be read as implying that less educated parents would find the consent process less deliberative or less coercive, only that this study cannot speak to how their experience, or the resources available to them, might differ. Furthermore, because all participants in this study were foreign-born, the sample consists exclusively of first-generation immigrant narratives. This study did not include US-born second-generation Turkish Muslim parents; therefore, findings cannot be directly transferred to or compared with second-generation parents who may possess different levels of institutional familiarity, language fluency, or cultural acculturation.

The same recruitment pathway most plausibly explains why the sample also skewed heavily male—eleven of thirteen participants. We cannot rule out that this partly reflects who, within these families, was available or willing to discuss vaccination decisions with unfamiliar interviewers; but a recruitment process built on professional and academic contacts is the more parsimonious explanation, and one this study is not positioned to disentangle from any substantive gendered pattern within the community itself.

A further limitation concerns the diversity of state regulatory contexts within our sample. Seven participants resided in Connecticut (10), and the remaining six in Massachusetts (26), New Jersey (27), Pennsylvania (11, 12), or Florida (28). With the exception of Connecticut, which eliminated its religious exemption for the 2022–23 school year, all of these states currently retain a religious exemption from school vaccination requirements. Vaccination-related legislation in each of these states has been subject to change in recent years, and our interviews did not record the specific school year in which each participant’s children were enrolled. We are therefore unable to establish which version of a given state’s law was in force at the time of each participant’s own experience; we describe each state’s regulatory context as it currently stands, rather than as a documented history of each participant’s individual encounter with it.

Despite these constraints on generalizability, the qualitative depth of the data—from participants navigating the same mandatory system with more resources than the populations most often studied in this literature—offers insight not otherwise available into how a mandate interacts with religious conviction and clinical communication, and provides a basis for the comparative, more broadly sampled research future studies should pursue.

Finally, the interview guide focused on routine childhood immunizations rather than COVID-19 vaccines; thus, the findings describe parents’ experiences within routine pediatric care and should not be interpreted as a direct measure of COVID-19 vaccine decision-making or pandemic-driven belief revision.

### 2.5 Ethical approval

We conducted the research in accordance with the principles of the Helsinki Declaration. The studies involving human participants were reviewed and approved on December 3, 2025, with approval number 2025-23/16, by the Bursa City Hospital Ethics Committee, an independent ethics committee appropriately established within the framework of the World Medical Association’s Helsinki Declaration (75th World Medical Association General Assembly, Helsinki, Finland, October 2024) and Good Clinical Practice Guidelines. We informed participants about the purpose, scope, confidentiality principles, and data usage of the research, and we obtained written informed consent from each participant. We anonymized all collected data and used it solely for scientific purposes.

## 3 Results

This study, utilizing a codebook thematic analysis of in-depth interviews conducted with Muslim Turkish parents living in the US, revealed four interrelated main themes shaping vaccination experiences: (1) Technical Language and Understandability Barriers in the Information Process, (2) Insufficiency in Addressing Cultural and Religious Sensitivities in Clinical Communication, (3) The Mandate of Consent Forms in Education and Enrollment Processes, (4) Information Verification Mechanisms. The findings for each theme and its sub-themes are presented below in the order shown. The themes and subthemes identified through the analysis are shown in Figure 2.

**Figure 2:**
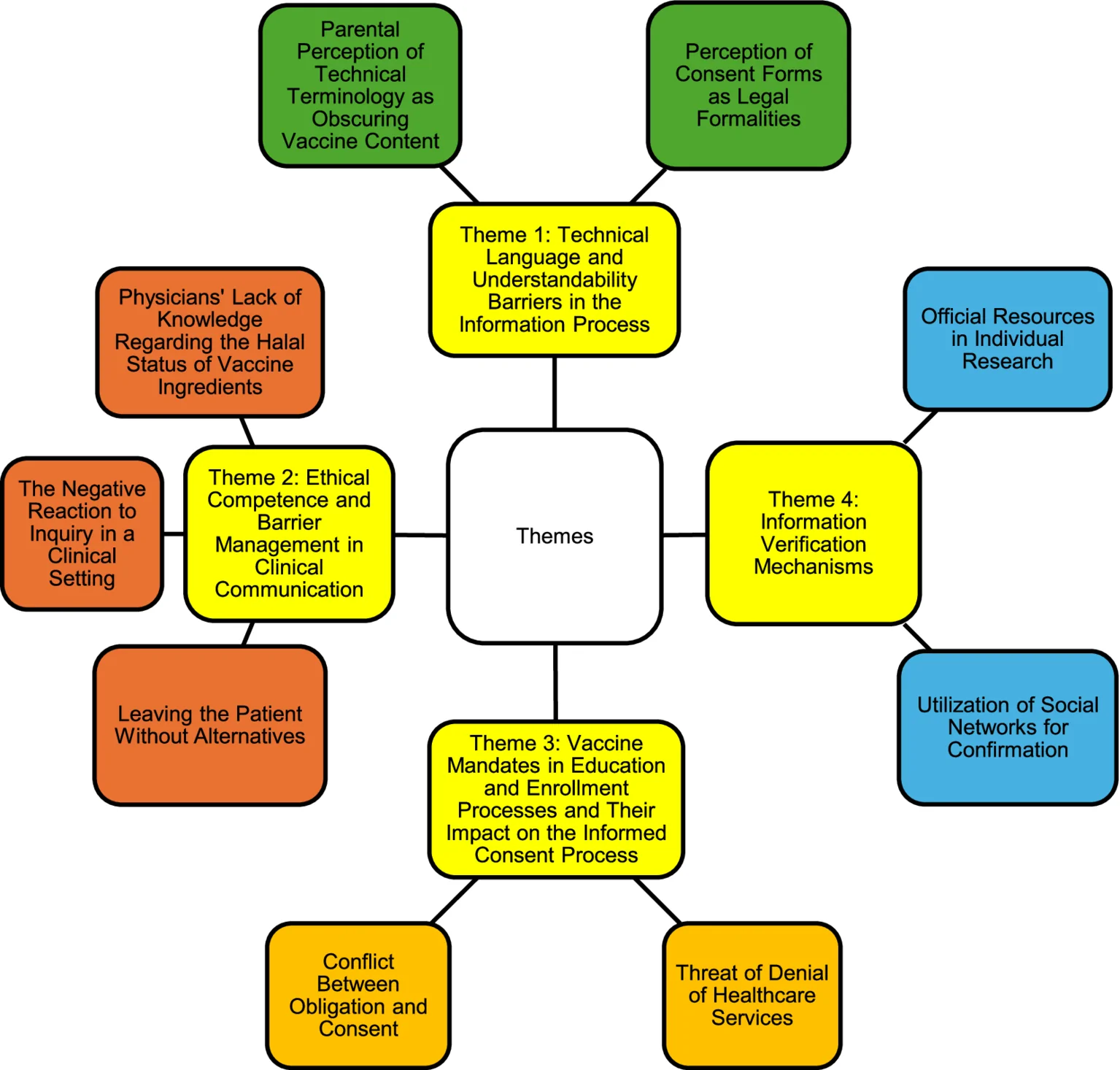
Conceptual map for the thematic analysis of vaccination information processes among Turkish parents in the United States.

### 3.1 Theme 1: technical language and understandability barriers in the information process

This theme addresses how the complexity of medical terminology and consent forms renders parents’ right to “be informed” ineffective. Presenting vaccine contents using technical names made a genuine risk analysis impossible for parents, and these documents were perceived not as educational tools but as documents intended to protect the institution’s legal responsibility.

#### 3.1.1 Parental perception of technical terminology as obscuring vaccine content

Participants stated that presenting vaccine ingredients (e.g., thimerosal) with technical names prevented the public from understanding their common names (e.g., mercury).

“*They tell you what’s in it, but I don’t know how much you can understand. For example, I didn’t know that thimerosal was a heavy metal. If you say thimerosal, there weren’t so many phones, Google, or information back then. What is thimerosal? The name sounds like an Italian pastry, but it’s mercury. I didn’t even know that mercury was so bad for human health.* (P6)”

#### 3.1.2 Perception of consent forms as legal formalities

Informed consent forms are perceived by parents not as educational tools, but rather as a legal procedure that protects the institution from liability. The length and complexity of the forms lead parents to treat them as mere formalities that are signed without being read.

“*I sign saying I’ve read and understood, and I don’t actually read it. Because it’s too long and unnecessary. The person is reading you something that could choke you, could kill you. They explain everything in detail so that legally nothing bad happens to you tomorrow or the day after.* (P13)”

### 3.2 Theme 2: ethical competence and barrier management in clinical communication

Participants described the perceived insufficiency of clinical communication regarding cultural and religious sensitivities as a primary gap in their informational encounter. Parents report that their questions about the content in clinical settings are met with surprise or that physicians lack sufficient knowledge on the subject.

#### 3.2.1 Physicians’ lack of knowledge regarding the halal status of vaccine ingredients

Participants reported perceiving that clinical providers were either uninformed about pork-derived ingredients or hesitant to discuss them during routine visits. In some cases, parents have warned physicians about the ingredients after conducting their own research. However, some parents avoid asking questions due to perceived insufficient expertise.

“*When the doctor says there’s no pork content, because I know, I ask if they can confirm it again, saying that this particular brand of vaccine from US has it. They check and say yes, it’s that brand, it’s written on the packaging. What if they hadn’t seen it?* (P3)”

*“I don’t know how much the doctor really knows. Because nurses usually administer the vaccines. If you ask a nurse, they’ll go and ask the doctor. The doctor will say, ‘I’m not sure.’” (P13)*

#### 3.2.2 The negative reaction to inquiry in a clinical setting

Parents stated that their ethical and religious inquiries about the medical content were met by physicians in the clinical setting as a strange situation encountered for the first time.

“*I remember when we said we cared a little about the content, we received a reaction as if we were encountering something like this for the first time.* (P12)”

#### 3.2.3 Leaving the patient without alternatives

The information phase of informed consent indicates that physicians did not provide participants with sufficient information about vaccine contents and alternatives.

“*In the clinic we went to, they generally only had one type of vaccine. Therefore, there was no instance in which they ordered a different vaccine for us.* (P12)”

### 3.3 Theme 3: vaccine mandates in education and enrollment processes and their impact on the informed consent process

Parents stated that they view the informed consent process not as a matter of medical preference, but as a formal requirement for accessing fundamental services such as the right to education. Participants explained that they were required to have their children vaccinated in order to complete the school enrollment process and remain in the system, and that they therefore viewed these consent forms as a necessity and signed them. Furthermore, parents noted that they were not offered alternative options regarding vaccinations and therefore had to approve the forms due to this lack of alternatives.

#### 3.3.1 Conflict between obligation and consent

The requirement for a “vaccination report” for school registration appears to conflict with the elements of “intent” and “free will” in the consent process. Parents view vaccination not as a medical benefit, but as a rule that must be fulfilled to remain part of the system, and they give their consent under this pressure.

“*When our children reach school age, a vaccination report is required for every school registration. There is a rule here. In most states across America, this is a standard rule. If it’s not up to date, they warn you that you have to remove your child from school if you don’t get it done within 15 days. There is a seriousness to it.* (P10)”

“*When you send your child to school, they ask for proof that you have had these vaccinations done, and the school may refuse you. In other words, they may not admit the child to the school.* (P5)”

Participants’ relationship to this rule was not uniform. P10 had declined one specific vaccine (MMR) for two of his children on safety grounds and had formalized this through the religious-exemption category available in a previous state of residence; after relocating to Connecticut, where that category is no longer recognized, school compliance warnings—rather than any change in his own risk assessment—led him to complete the vaccination. P5, who reported no hesitation toward any vaccine, described the same registration requirement in purely procedural terms. Read together, these accounts suggest that the exemption categories offered by the state do not necessarily track parents’ actual reasoning, and that what resolves non-compliance can be administrative pressure rather than a shift in underlying conviction.

#### 3.3.2 Threat of denial of healthcare services

Some participants stated that in the US, some healthcare organizations have excluded parents who refuse vaccination from services. On the other hand, participants described experiencing mandatory school vaccination requirements as a procedural prerequisite necessary to secure educational access rather than a voluntary medical decision.

“*In the US, they don’t want to treat people who don’t get vaccinated. For whatever reason, they don’t treat them.* (P3)”

“*In the first few years after I arrived, they really pressured us to get vaccinated. They even imposed sanctions back then.* (P1)”

“*It will come up again in some way in the future. We said, ‘Let’s go and get this vaccine, let’s get rid of this problem.’* (P10)”

### 3.4 Theme 4: information verification mechanisms

Parents stated that they seek alternative channels to manage their own consent processes. Participants noted that they utilize artificial intelligence, official institutional websites, and digital platforms as alternative information sources to access technical details and religious suitability information that they did not obtain from physicians.

#### 3.4.1 Official resources in individual research

Parents are conducting individual research using internet search engines and AI-based tools to address information gaps and communication issues in the traditional healthcare system. Some parents are trying to overcome this process primarily through the official websites of institutions and organizations.

“*Here we have the FDA (Food and Drug Administration). They list the contents of all the different vaccine brands licensed in America on their website. You can access their package inserts.* (P12)”

“*The Centers for Disease Control and Prevention (CDC) generally recommends these. The CDC is called the Centers for Disease Control and Prevention. You can get the schedule from there.* (P10)”

#### 3.4.2 Utilization of social networks for confirmation

For some parents, where trust in doctors is shaken, sharing experiences within their social circle and “community” takes on a higher hierarchy of trust than formal medical information. Parents fill systemic information gaps by receiving clinically sensitive advice, tailored to their beliefs, through these social networks.

“*I asked (Generative Pre-trained Transformer 3.5) ChatGPT… they commented that if there is no alternative in a necessary situation, nothing can be done through medical necessity.* (P2)”

“*Generally, on social media, I listen to a lot of podcasts… We listen to doctors and people close to doctors who have developed themselves.* (P6)”

“*There was a website by a professor in Türkiye, I read a bit of it. It said that even if you don’t get all the others, you should get these things done*. (P11)”

Family and social environment are among the key determinants shaping participants’ attitudes toward vaccination. Especially for parents who have healthcare professionals in their family, these individuals serve as a means of blending medical knowledge with cultural values.

“*We try to use food, drinks, and medicines by asking each other about halal and haram aspects, and researching them on various platforms. We try our best to be sensitive in that regard.* (P11)”

“My father is a doctor, by the way. He told me, ‘Son, these vaccines are important for children and need to be done, but absolutely do not get any extra vaccines.” (P13)

Furthermore, the intense exchange of ideas and sharing of experiences within the community creates both a support system and a validation mechanism for the participants, as this social network plays a significant role in their decision-making process.

“*I asked my friends in the community, ‘Where should I take the baby, what should I do?’ What are your stories? You hear a different story from each of them.* (P13)”

“*Before we went to this doctor, and this is where the importance of our community comes into play, they said that if you go to this clinic, they are sensitive about this issue. They definitely use animal products, but no pork, they said. So we went there.* (P13)”

## 4 Discussion

Across four interconnected findings, our results show that technical medical terminology obscured rather than clarified vaccine-related information for parents, that physicians were frequently unable to address religious and cultural concerns such as the halal status of vaccine ingredients, that consent forms functioned less as a deliberative tool than as an administrative requirement tied to school enrollment, and that parents responded by independently verifying information through official sources, community networks, and AI tools. Taken together, these findings indicate that highly educated Turkish parents living in the US experience the informed consent process not as an ethical decision-making stage, but as a bureaucratic formality—one in which mandatory vaccination requirements imposed at school registration, combined with the absence of meaningful alternatives, undermine the autonomous and informed nature of consent.

For most participants, the consent experience was shaped less by what was explained to them than by the fact that non-compliance was not framed as a realistic option. Connecticut, where the majority of participants resided, has not recognized a religious basis for exemption from school vaccination requirements since the 2022–23 school year (10); Pennsylvania, where one participant resided, retains one in statute (11, 12). This legal distinction, however, may matter less than it first appears: one participant’s decision to comply in Connecticut was driven not by a change in his own risk assessment but by school compliance pressure, after the religious-exemption category he had used in a previous state was no longer available to him—suggesting that the category the mandate turns on did not track the reasoning it was meant to accommodate. Assessed against Savulescu’s four-condition framework for justifiable mandatory vaccination—a grave threat to public health, a safe and effective vaccine, a favorable benefit–cost ratio relative to less restrictive alternatives, and coercion proportionate to that threat (25)—the Connecticut requirement, in our assessment, plausibly satisfies the first three conditions; whether it satisfies the fourth cannot be established from our data, since our participants’ accounts do not show whether the exemption structure available to them tracked their actual convictions. Sophian et al. (2026) make a related point, arguing that policy shifts of this kind are read internationally less as evidence-based public health decisions than as signals about institutional trust (16)—consistent with how participants in our study described the requirement more generally, one recalling that continued resistance was not worth pursuing once, in his words, “it will come up again in some way in the future” (P10). The cost of non-compliance extended further for some participants, consistent with the broader literature on stigma and vaccine refusal: one described being refused treatment outright, another described being pressured and, in earlier years, sanctioned (P3, P1) (29, 30).

This tension between legal permissibility and ethical adequacy is not merely theoretical. Yang and Olick, writing on the wave of state-level religious-exemption repeals of which Connecticut’s is one instance, note that these policies remain publicly and judicially contested even as they raise vaccination rates (31). Miller v. McDonald—in which Amish families and schools challenged New York’s 2019 elimination of religious exemptions, the same policy structure now in force in Connecticut, as a violation of the Free Exercise Clause—illustrates this tension at the constitutional level; amici in the case argue that a regime permitting medical but not religious exemptions treats a burden on the body as more legitimate than a burden on conscience. The Supreme Court subsequently vacated and remanded the case for further review, and on remand the Second Circuit again upheld the mandate in June 2026, the most recent ruling in ongoing litigation (32). Whatever its ultimate resolution, this litigation settles only the constitutional question, not the ethical one our participants’ accounts raise: whether a consent process embedded in a mandate that permits no accommodation for religious conviction can still be meaningfully called voluntary. The American Academy of Pediatrics has itself endorsed eliminating non-medical exemptions on public health grounds while urging pediatricians to keep counseling families who seek them (14), a policy stance that speaks to clinical practice but leaves the same question unresolved.

The consent parents give for their children to be vaccinated in order to meet school enrollment requirements is only part of the picture; the real issue is whether the consent obtained during the clinic visit—that is, at the appointment where the vaccine is actually administered—meets the criteria for a valid informed consent process in and of itself, independent of any requirement. Jagadish et al. (5), building on the Faden and Beauchamp model of autonomous decision-making, argue that a legitimate consent process must be more than a signature stage: it must engage a parent’s competence, intentionality, and substantial understanding of the material facts, free from controlling influence. Participants’ accounts suggest the understanding condition, in particular, was frequently unmet—not because parents were pressured to sign, but because the clinical encounter did not equip them to engage with what they were signing. Technical terminology obscured rather than clarified risk for at least one participant, who did not recognize the term thimerosal—which has no evident link to mercury—as the substance he associated with mercury exposure and would otherwise have wanted explained (P6). We report this as the participant’s own understanding rather than a scientific claim: thimerosal is a mercury-containing organic compound used as a preservative in some vaccines, not mercury itself or “a heavy metal” in the strict sense, and our interview data do not indicate which specific vaccine, or which stage of production, the participant had in mind. Another described the written consent form as a repeated formality rather than a deliberative step: a lengthy document that he signed as “read and understood” without reading it, because a verbal recitation of risk—delivered, in his account, “so that legally nothing bad happens to you tomorrow or the day after”—had already stood in for it (P13). This pattern—thorough in its liability disclosures but not evidently organized around what a parent could absorb—is consistent with Reiss and Karako-Eyal’s review of the informed-consent readability literature, which finds that shortening a form does not reliably improve comprehension unless the underlying disclosure itself changes (33). What both accounts describe is a process organized around institutional liability rather than parental understanding.

A comparable gap in understanding appeared around a question specific to this population: the religious permissibility of vaccine ingredients. Physicians were sometimes unable to confirm, or initially unaware, whether a given vaccine contained porcine-derived components—information one participant had to supply after independent research (P3)—and inquiries about halal status were at times met with visible surprise rather than a ready answer, leaving families without any discussion of alternatives (P12, P13). This is consistent with Durmaz et al.’s finding that the large majority of pediatricians in Turkey report needing further training in clinical ethics generally (34), and with Jinarong et al.’s argument that physicians who cannot engage a family’s religious framework leave parents to resolve such questions elsewhere (35). None of this required a mandate to produce: it reflects the limits of what physicians offered, not pressure on what parents were made to accept. The underlying question, though, is the same one at stake in Connecticut’s registration requirement: Smith and Mackie argue that a legal vaccination requirement does not, on its own, make the resulting consent involuntary, provided the surrounding process still meets ordinary standards of understanding and non-manipulation (36); Grzybowski et al. (37) draw a related distinction between coercion that bypasses a person’s reasoning and a constraint that still leaves room for a reasoned decision. Taken together, these accounts suggest two independent and compounding problems rather than substitutes for one another: whether or not Connecticut’s mandate satisfies Savulescu’s proportionality condition—a question our data cannot resolve—a mandate that did would still not, by itself, guarantee an autonomous decision in Faden and Beauchamp’s sense, because the clinical encounter can fail the understanding condition regardless of what the school requires. Parents in our study faced both a registration requirement that left little room to decline and a clinical process that gave them, in practice, limited basis on which to have chosen otherwise.

Individual clinician knowledge gaps are not the whole explanation for why halal status so often went unaddressed: much of what a physician can offer depends on guidance that exists—or does not exist—well upstream of any single clinical encounter. Where religious authorities and manufacturers coordinate, the halal-status question can be resolved before it ever reaches an individual clinical encounter. The European Council for Fatwa and Research ruled in 2003 that trace porcine trypsin in polio vaccines could be used under the doctrine of necessity (darurah), reasoning that withholding the vaccine would cause greater harm than the impermissible ingredient itself—a general jurisprudential answer that spared physicians and families from resolving the question individually (38). The same doctrine has since been applied at a far larger scale: Indonesia’s Ulema Council ruled in March 2021 that the AstraZeneca COVID-19 vaccine, though haram for its use of porcine-derived trypsin in manufacturing, was nonetheless “haram-permissible” given the pandemic’s urgency and the absence of an alternative, and the United Arab Emirates’ Fatwa Council reached a comparable conclusion on the same grounds (39). Malaysia’s MS 2424:2019 halal-pharmaceutical standard operationalizes this further: Zulkarnain et al. (40) work through a standard influenza vaccine ingredient by ingredient and identify sharia-compliant substitutes for porcine trypsin and gelatin-based stabilizers, while noting that alternatives for other components—including the host cell line and growth media—do not yet fully satisfy sharia requirements given current manufacturing constraints. None of this required a treating physician to know or explain the underlying chemistry; a jurisprudential ruling and a national manufacturing standard had already done that work in advance, even where that work remained incomplete.

This kind of coordination is not automatic, and its absence carries a measurable cost. When a provincial Indonesian Ulema Council publicly questioned the halal status of the measles-rubella vaccine in 2018, the resulting confusion—even after the central council permitted the vaccine’s use on the same necessity grounds—left an estimated 10 million children uninoculated in the campaign’s second phase (41), evidence that the gap between religious uncertainty and public health outcomes is not merely theoretical. Comparable religious guidance does in fact exist within the US: the Fiqh Council of North America and the Assembly of Muslim Jurists of America have each issued rulings on the permissibility of specific vaccines—including, in AMJA’s case, a ruling addressing porcine-derived gelatin in routine childhood vaccines such as MMR—reasoning in terms consistent with the same necessity doctrine (darurah) (42, 43). Our interviews, however, did not establish whether participants were aware of, sought, or received guidance from these or other US-based Islamic authorities, or whether such guidance reached the physicians our participants saw. None of our participants described consulting a formal religious-legal ruling of this kind; instead, they relied on informal substitutes of their own. One participant described his father, himself a physician, advising him that the required vaccines were important but that he should seek nothing beyond them (P13); another recounted that before choosing a clinic, community members described it as “sensitive about this issue,” noting that it “definitely use[d] animal products, but no pork”—which is why they went there (P13); a third described checking halal and haram content by “asking each other” and researching it “on various platforms” rather than relying on a clinician (P11). This is broadly consistent with Alsuwaidi et al.’s observation that halal labeling and the guidance of religious leaders, more than the clinical encounter itself, shape Muslim parents’ vaccination decisions (44). Whether a family’s physician happens to be, as one participant put it, “sensitive about this issue,” becomes a matter of social connection rather than a predictable feature of the healthcare system—a structural gap that individual clinician training, however improved, cannot close on its own.

Our sample’s gender composition—eleven of thirteen participants were fathers—likely shaped not only who reported these experiences but what surfaced in them. Most qualitative research on vaccine hesitancy centers mothers as the presumed primary healthcare decision-makers, treating the absence of fathers’ perspectives as the limitation to note (45); our study inverts that pattern without resolving the same underlying gap. Whether mothers in these same families navigated the school mandate, the clinical encounter, or the search for halal guidance differently—through different networks, different leverage, or different stakes—is a question this study is not positioned to answer. The findings in this study should accordingly be read as describing how these fathers experienced and narrated the process, not as a general account of Turkish Muslim parents’ experience.

Parents’ recourse to official databases such as the FDA and CDC did not arise in isolation: several participants linked it directly to gaps in physician communication regarding vaccine ingredients and halal status, seeking on their own the specificity their clinical visits did not supply. This individual review of official institutional data (P12, P10) is consistent with what the broader literature calls the “searcher” profile—parents who seek vaccine information primarily from published sources rather than relying on social norms or others’ advice (46, 47). This self-directed research served a legitimate purpose, filling gaps physicians left open; but it carried its own risk, since neither AI tools nor internet searches offer the same accountability as a clinical relationship, and information gathered this way can be mistaken for understanding without being checked against it. The volume and inconsistency of vaccine-related information available online compounds this risk, sometimes described in the literature as an “infodemic”—an excess of information, not all of it reliable, that can leave parents less rather than more able to distinguish sound guidance from misinformation (48, 49). Digital tools of this kind can support the understanding component of informed consent, but they cannot substitute for a clinical relationship capable of the empathy and cultural sensitivity vaccine decisions in this population require (5, 16).

While our semi-structured interview protocol focused specifically on routine childhood immunizations rather than COVID-19 vaccines, the broader societal context of the COVID-19 pandemic inevitably forms a backdrop to participants’ information practices. The pandemic triggered a global surge in vaccine-related discourse, marked by heightened institutional mistrust, widespread online misinformation, and what public health authorities termed an ‘infodemic’ (48, 49). Existing literature demonstrates that the pandemic era accelerated parental self-directed digital information-seeking and intensified public scrutiny over vaccine safety and ingredient transparency (30, 45). However, because our interviews did not explicitly evaluate COVID-19 vaccine decision-making, we do not suggest that the pandemic directly altered the foundational religious or ethical convictions of these participants. Rather, the pandemic-era informational environment likely amplified pre-existing tendencies toward independent digital verification and heightened sensitivity to clinical communication gaps.

## 5 Conclusion

In contrast to the common focus on low literacy and passive recipient status in refugee studies, this research reveals that participants developed active validation mechanisms to reach an autonomous and informed vaccination decision.

In conclusion, this study offers an in-depth account, from a small and specific sample, of how thirteen first-generation Turkish Muslim parents experienced informed consent for their children’s vaccinations. Their accounts point to three distinct, compounding difficulties: a mandate that left little visible room for individual religious commitments; a clinical encounter that, independent of the mandate, often did not equip parents to engage meaningfully with what they were signing; and, for the specific question of halal status, a reliance on informal community and family networks in place of the formal religious guidance that exists in the US but that none of our participants described consulting. Better clinician training and communication may narrow the gap in the clinical encounter, and closer coordination between healthcare systems, manufacturers, and US-based Islamic authorities may narrow the systemic one; our data cannot establish that either would succeed, only that our participants’ difficulties point in these directions. Because our sample was limited to thirteen first-generation immigrants, primarily fathers, all with at least a bachelor’s degree and recruited through a connected professional network, these findings should not be generalized to US-born Turkish Muslims, mothers, parents with different educational backgrounds, or families outside this network. Future research, including comparative and longitudinal work, would be needed before any of these possibilities could be treated as policy recommendations. Future comparative studies are recommended to investigate potential generational differences by systematically comparing foreign-born first-generation parents with US-born second-generation Turkish Muslim parents regarding their health information practices, vaccine decision-making, and perceptions of clinical informed consent.

## Data Availability

The original contributions presented in the study are included in the article; further inquiries can be directed to the corresponding author, while raw transcripts are restricted due to participant privacy and confidentiality.
